# Rosiglitazone diminishes the high-glucose-induced modulation of 5-fluorouracil cytotoxicity in colorectal cancer cells

**DOI:** 10.17179/excli2018-1011

**Published:** 2018-02-06

**Authors:** Meng-Fei Lau, Shalini Vellasamy, Kek-Heng Chua, Vikineswary Sabaratnam, Umah Rani Kuppusamy

**Affiliations:** 1 Department of Biomedical Science, Faculty of Medicine, University of Malaya, 50603 Kuala Lumpur, Malaysia; 2 Mushroom Research Centre, University of Malaya, 50603 Kuala Lumpur, Malaysia; 3 Institute of Biological Science, Faculty of Science, University of Malaya, 50603 Kuala Lumpur, Malaysia

**Keywords:** rosiglitazone, 5-fluorouracil, high glucose, MTT, cell cycle, colorectal cancer cell

## Abstract

Colorectal cancer (CRC) is the third most leading cause of morbidity and mortality throughout the world. 5-fluorouracil (5-FU), which is often administrated to disrupt carcinogenesis, was found to elevate blood glucose level among CRC patients. Thus, this study was conducted to evaluate the influence of rosiglitazone on antiproliferative effect of 5-FU using cellular model. Two human colonic carcinoma cell lines (HCT 116 and HT 29) were cultured in the presence of 5-FU, rosiglitazone or in combination under normal and high glucose concentration. The drug cytotoxicity was evaluated using the MTT assay whereas the assessment of cell cycle was carried out using the flow cytometry technique. Combination index (CI) method was used to determine the drug interaction between rosiglitazone and 5-FU. High glucose diminished the cytotoxic effect of 5-FU but at a high drug dosage, this effect could be overcome. Cell cycle analysis demonstrated that 5-FU and rosiglitazone caused G1-phase arrest and S-phase arrest, respectively. CI values indicated that rosiglitazone exerted synergistic effect on 5-FU regardless of glucose levels. This study is the first to demonstrate the influence of rosiglitazone on cytotoxicity of 5-FU under normal or high glucose level. Rosiglitazone may be a promising drug for enhancing the efficacy of 5-FU in the treatment of CRC associated with hyperglycemia.

## Introduction

Colorectal cancer (CRC) is the third most leading cause of morbidity and mortality throughout the world (Ferlay et al., 2015[13]). It is estimated that over one million new cases are diagnosed per year, which accounts for around 10 % of all cancer incidences in both sexes.

Chemotherapy with curative intent is often applied as a first-line treatment to disrupt carcinogenesis. In this context, 5-fluorouracil (5-FU) plus leucovorin is the main option for CRC patients (Goodwin and Asmis, 2009[17]). Other active agents such as irinotecan, capecitabine, oxaliplatin, bevacizumab, cetuximab or panitumumab are also adopted in fluorouracil-based therapy to improve overall survival and reduce the risk of disease recurrence. Unfortunately, the clinical efficacy is highly dose-dependent due to pharmacokinetic variability and it causes severe toxicities in some CRC patients despite the administration of standard drug protocol (André et al., 2004[2]).

Based on previous epidemiological studies, diabetes mellitus is suggested to be an independent risk factor for colorectal cancer. Although the relationship is not entirely understood, most findings concur with a positive association between diabetes and colorectal cancer (de Bruijn et al., 2013[8]; Deng et al., 2012[9]; Krämer et al., 2012[23]; Larsson et al., 2005[24]; Luo et al., 2012[29]; Mills et al., 2013[35]). Giovannucci et al. (2010[16]) reported that plausible biological mechanisms underlying this association can be attributed to the effect of hyperglycemia, hyperinsulinemia or inflammation on cancer aetiology and progression. An improved glucose control should be an important therapeutic approach for CRC patients. 

Besides metformin, thiazolidinediones including rosiglitazone and pioglitazone are another class of oral antidiabetic drugs which help to protect against hyperglycemia (Yki-Järvinen, 2004[46]). Thiazolidinediones increase insulin sensitivity by activating one or more peroxisome proliferator-activated receptors (PPARs) to regulate glucose utilization and production. Several *in vitro* studies indicated that rosiglitazone acts as a PPAR-gamma agonist which suppresses cell proliferation, inhibits cell invasiveness, arrests cell cycle and induces apoptosis in cancer cell lines (Cao et al., 2009[4], 2015[5]; Han and Roman, 2006[18]; He et al., 2008[20]; Lin et al., 2007[25]; Zhang et al., 2008[49]). These anticancer properties provoke the use of rosiglitazone on individuals who developed diabetic complications during or after fluorouracil-based regimen. 

A recent meta-analysis showed a decreased risk of colorectal cancer incidence when rosiglitazone was administered to diabetic patients (Monami et al., 2014[36]). While high glucose can modulate cytotoxicity of 5-FU (Ma et al., 2014[30]), the efficacy of rosiglitazone is possibly affected by the level of hyperglycemic condition. In this study, the influence of rosiglitazone on 5-FU pretreated human colon cancer cell lines was evaluated at high glucose level. The drug interaction between rosiglitazone and 5-FU was also determined.

## Material and Methods

### Reagents

Blank glucose Dulbecco's Modified Eagle Medium (DMEM) (Cat. no.: 11966) and normal glucose DMEM (Cat. no: 11885) were purchased from ThermoFisher. High glucose DMEM (D5671 Sigma) was complemented with 4.0 mM L-glutamine, 1.0 mM sodium pyruvate, 10 % fetal bovine serum, 1 % penicillin streptomycin and 1 % amphoterin B. The stock solutions of 5-FU (F6627 Sigma) and rosiglitazone (R2408 Sigma) were prepared in phosphate-buffered saline (PBS) and dimethyl sulfoxide (DMSO) with a concentration of 5 mg/ml and 1 mg/ml respectively. MTT formazan powder (M5655 Sigma) was dissolved in PBS and diluted to 5 mg/ml. Propidium iodide (PI, Cat. no: P1304MP) and RNAse A (12091-021) were obtained from ThermoFisher. 

### Cell culture

Two human colonic carcinoma cell lines, namely HCT 116 and HT 29, were cultured in normal glucose DMEM (Cat No: 11885) supplemented with 10 % fetal bovine serum, 1 % penicillin streptomycin and 1% amphoterin B. A human normal colon cell line (CCD-18Co) was cultured in normal glucose Minimum Essential Medium Eagle (MEME, D2279 Sigma) complemented with 4.0 mM L-glutamine, 1.0 mM sodium pyruvate, 20 % fetal bovine serum, 1 % non-essential amino acid and 1 % penicillin streptomycin. All cell lines were maintained in humidified atmosphere of 5 % CO_2_ at 37±2 °C.

### Cell proliferation assay

HCT 116 cells, HT 29 cells and CCD-18Co cells were seeded into 96-well plates at a density of 2000 cells/100 µl, 2500 cells/100 µl and 3500 cells/100 µl per well respectively. After incubation at 37±2 °C for 24 h, the cells were then treated with 5-FU or rosiglitazone at various concentrations for 48 h. Next, 10 µl of MTT was added and the plates were incubated in the dark at 37±2 °C for 3 h. After solubilizing the formazan crystals in 100 µl DMSO, absorbance was measured at 560 nm. The cell proliferation was calculated as follows: {[absorbance of treated group-absorbance of blank] ∕ [absorbance of control group-absorbance of blank]} × 100. The IC_20 _and IC_50_ were determined through linear regression analysis.

### Cell cycle assay

For the cell cycle assay, drug treatment was carried out in 12-well culture plates with a density of 40, 000 cells/ml/well (HCT 116) and 55, 000 cells/ml/well (HT 29) at 37±2°C for 48 h. The cells were harvested and fixed in 2 ml of cold 70 % (v/v) absolute ethanol and stored overnight at -20 °C. Then, the fixed cells were washed twice with PBS. Cell staining was done by sequentially adding 450 µl PBS, 25 µl RNAse A (1 mg/ml) and 50 µl PI (0.1 mg/ml). The stained cells were incubated in the dark at room temperature (25±2 °C) for 30 min and further examined by a flow cytometer (BD FACScanto II) integrated with BD FACSDiva Software. Modfit LT 2.0 was used to analyze DNA content histograms.

### Experimental design

First, the cell proliferation of HCT 116 and HT 29 under normal glucose (NG, 5.5mM) and high glucose (HG, 25mM) culture conditions for 48 h was carried out. The complete blank glucose DMEM containing 1.0 mM sodium pyruvate, 10 % fetal bovine serum, 1 % penicillin streptomycin and 1 % amphoterin B served as a negative control whereas NG DMEM+19.5 mM D-mannitol and blank glucose DMEM+25 mM D-mannitol were the osmotic controls. The cell proliferation of CCD-18Co cells was evaluated at different glucose concentrations with NG MEME+19.5 mM D-mannitol as an osmotic control.

To study the effect of glucose on drug cytotoxicity, both cancer and normal cells lines were pre-treated with 0.2 µg/ml 5-FU for 24 h. Then, spent culture media was aspirated and replaced with the following experimental media: (i)NG medium+0.2 µg/ml 5-FU; (ii)NG medium+2 µg/ml 5-FU; (iii)HG medium+0.2 µg/ml 5FU; (iv)HG medium+2 µg/ml 5-FU. The control group was 5-FU pre-treated cells in NG medium without any drug treatment for subsequent 48 h incubation. The same experiment was repeated with treatments of rosiglitazone alone and a combination of 5-FU and rosiglitazone. In this study, low dose was defined as a concentration close to IC_20 _of the drug (Kashif et al., 2015[21]). 

### Statistical analysis

SPSS Statistics 17.0 software, ANOVA followed by Duncan Multiple Range Test was performed to analyse the data. Value of p< 0.05 was considered statistically significant. All results were expressed as mean±SEM from three independent experiments of at least three replicates. 

## Results

### Effect of 5-FU and rosiglitazone on cell proliferation

**Figure 1:**
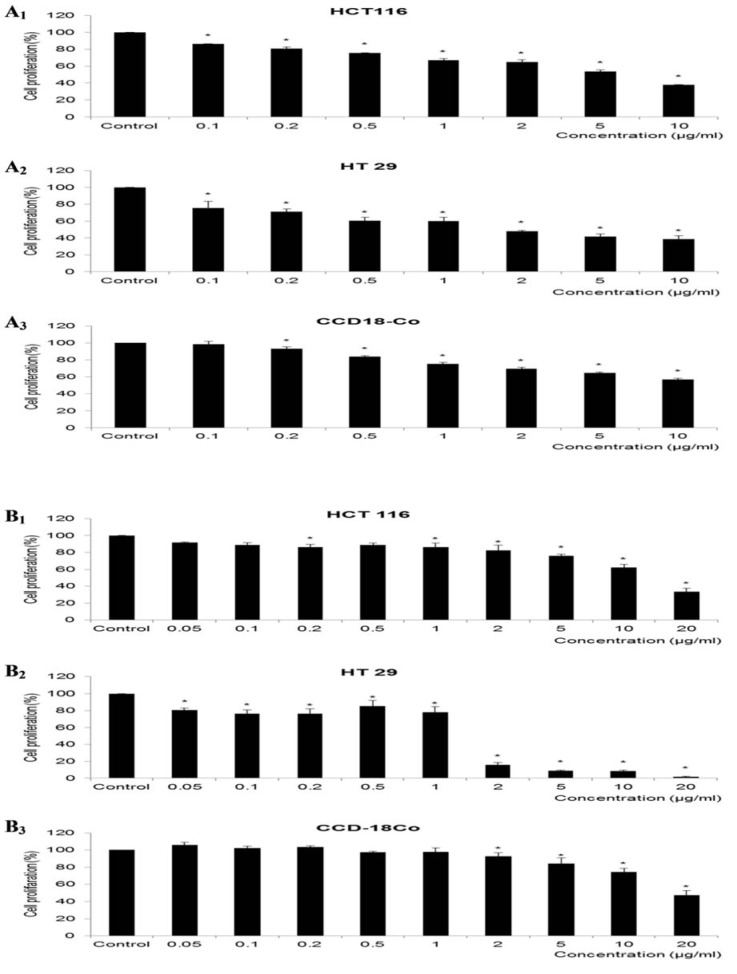
Effect of drug treatment on the cell proliferations. Cells were treated with (A) 5-FU or (B) rosiglitazone at various concentrations for 48 h. The control group were untreated cells in normal glucose medium. *Compared to control, p <0.05.

Figure 1(Fig. 1) shows the cell proliferation of HCT 116 and HT 29 cells upon treatment with increasing concentrations of 5-FU or rosiglitazone. Both cell lines showed a dose-dependent decrease in cell proliferation. HCT 116 responded to 5-FU (Figure 1A_1_(Fig. 1)) at IC_50_= 4.77±0.55 µg/ml and rosiglitazone (Figure 1B_1_(Fig. 1)) at IC_50_= 11.61±3.49 µg/ml. The respective drugs resulted in lower IC_50 _values of 1.86±0.18 µg/ml (5-FU, Figure 1A_2_(Fig. 1)) and 1.04±0.14 µg/ml (rosiglitazone, Figure 1B_2_(Fig. 1)) on HT 29. Since the IC_20_ of both drugs was comparable between HCT 116 and HT 29, a low dose of 5-FU at 0.2 µg/ml and rosiglitazone at 0.5 µg/ml were selected. 5-FU and rosiglitazone did not exert any significant effect at the low dose range but showed 30-40 % inhibition at extremely high concentration (>10 µg/ml) on CCD-18Co cells (Figures 1A_3_ and 1B_3_(Fig. 1)).

**Figure 2:**
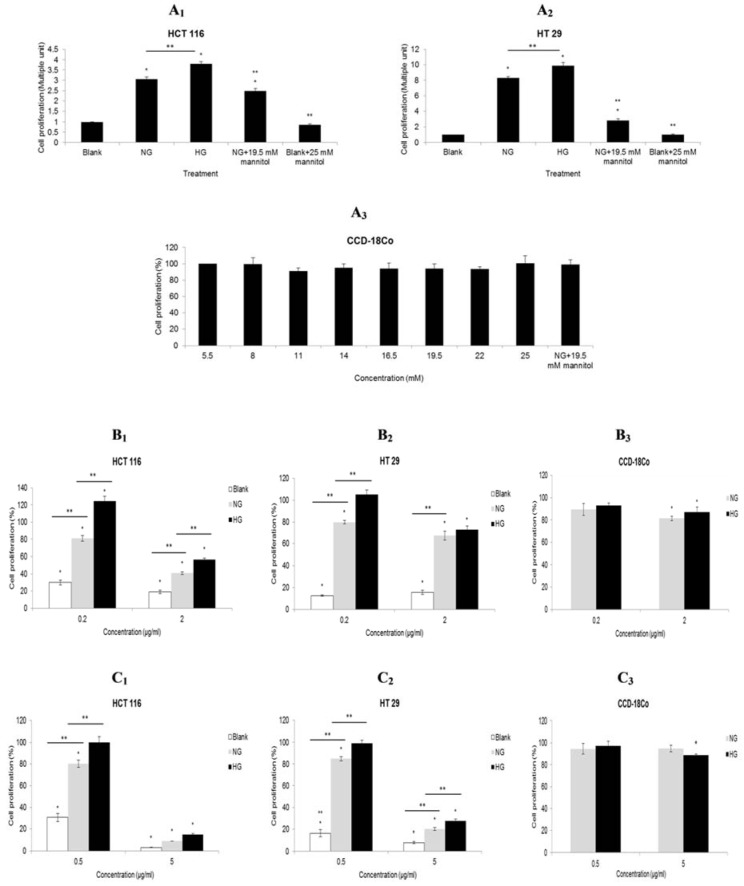
Effect of glucose on drug treatments. (A) HCT 116 and HT29 cells were treated in NG or HG culture for 48 h. The control group were cells incubated in blank medium. Two media containing mannitol with a concentration of iso-osmolar to HG was used as osmotic controls. CCD-18Co cells were cultured at different glucose concentrations. (B-C) 5-FU pretreated cells with subsequent low dose or high dose of drug treatments in blank (absent on CCD-18Co), NG and HG media for 48 h. The control group were 5-FU pre-treated cells in NG culture without any subsequent drug treatments. *Compared to control, p <0.05. **Compared to NG group, p <0.05. NG, normal glucose (5.5 mM); HG, high glucose (25 mM) Panel B: 5-FU, low dose =0.2 µg/ml; high dose =2 µg/ml, Panel C: Rosiglitazone, low dose =0.5 µg/ml; high dose =5 µg/ml

In HG culture, the cell proliferation was stimulated significantly 0.7 fold (HCT116, Figure 2A_1_(Fig. 2)) and 1.6 fold (HT29, Figure 2A_2_(Fig. 2)) compared with NG culture. Subsequent introduction of high glucose (25 mM) even reduced the inhibitory effect of 5-FU.

At low dose of 5-FU (0.2 µg/ml), HG culture significantly (p< 0.05) increased the cell proliferation of HCT 116 up to 124 % (Figure 2B_1_(Fig. 2)). The cell proliferation of HT 29 was approximately 105 % in HG culture and it was significantly (p< 0.05) higher than that in NG culture (80 %) (Figure 2B_2_(Fig. 2)). At low dose of rosiglitazone (0.5 µg/ml), a significant difference (p< 0.05) in cell proliferations between HG culture (HCT 116= 100%; HT 29= 99 %) and NG culture (HCT 116= 80 %; HT 29= 85 %) was evident (Figures 2C_1_ and 2C_2_(Fig. 2)). The viability of both cell lines was less than 30 %, either in HG or NG culture, when treated with a high dose of rosiglitazone (5 µg/ml). Glucose level had no significant effect on CCD-18Co cells regardless of drug treatment (Figures 2B_3_ and 2C_3_(Fig. 2)).

**Figure 3:**
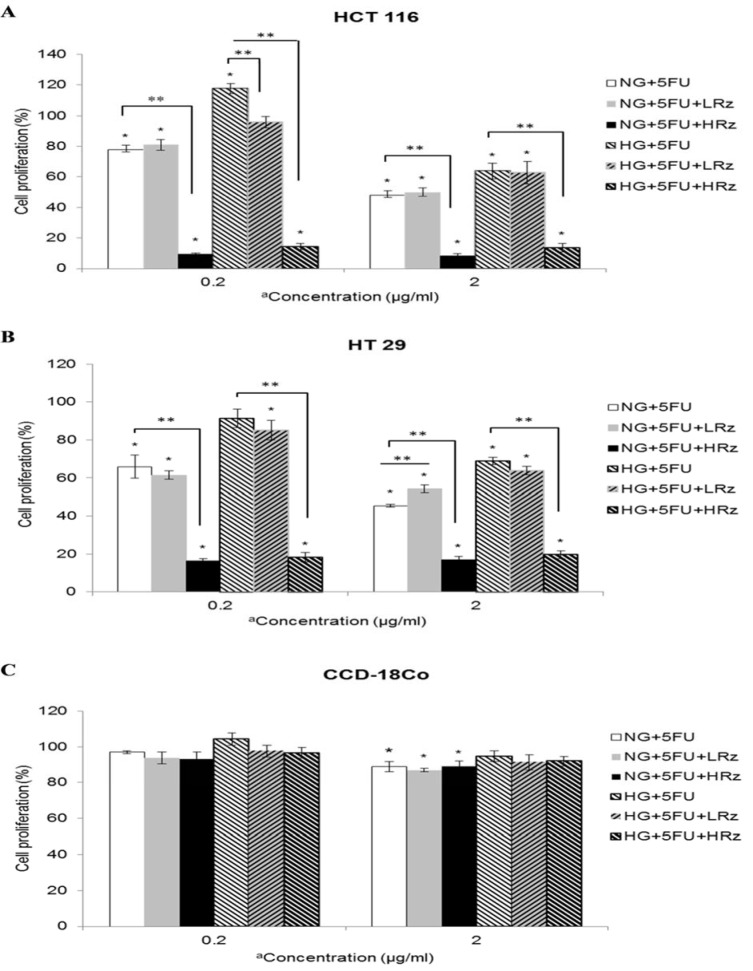
Effect of combined drugs on cell proliferation under NG or HG culture. 5-FU pretreated cells were further exposed to 5-FU alone or in combination with rosiglitazone for 48 h incubation. The control group were 5-FU pre-treated cells in NG culture without any subsequent drug treatments. *Compared to control, p <0.05. **Compared to single dose 5-FU, p <0.05. NG, normal glucose (5.5 mM); HG, high glucose (25 mM) ^a^0.2 µg/ml as low dose 5-FU; 2 µg/ml as high dose 5-FU LRz, low dose rosiglitazone= 0.5 µg/ml HRz, high dose rosiglitazone= 5 µg/ml

Figures 3A and 3B(Fig. 3) show the cell proliferations of HCT 116 and HT 29 respectively when 5-FU and rosiglitazone were used in combination. At low dose of 5-FU, an addition of low dose rosiglitazone significantly (p< 0.05) decreased the cell proliferation of HCT 116 from 118 % to 96 % in HG culture while the same treatment had no effect on HT 29. At high dose of 5-FU, an addition of low dose rosiglitazone in HG culture triggered the cells to proliferate at an equivalent rate to those treated with single dose of 5-FU. The two cell lines maintained viability below 20 % with combined drugs containing high dose of rosiglitazone in NG and HG cultures. Similarly, glucose level had no significant effect on CCD-18Co cells regardless of drug treatment (Figure 3C(Fig. 3)).

### Effect of 5-FU and rosiglitazone on cell cycle

**Figure 4:**
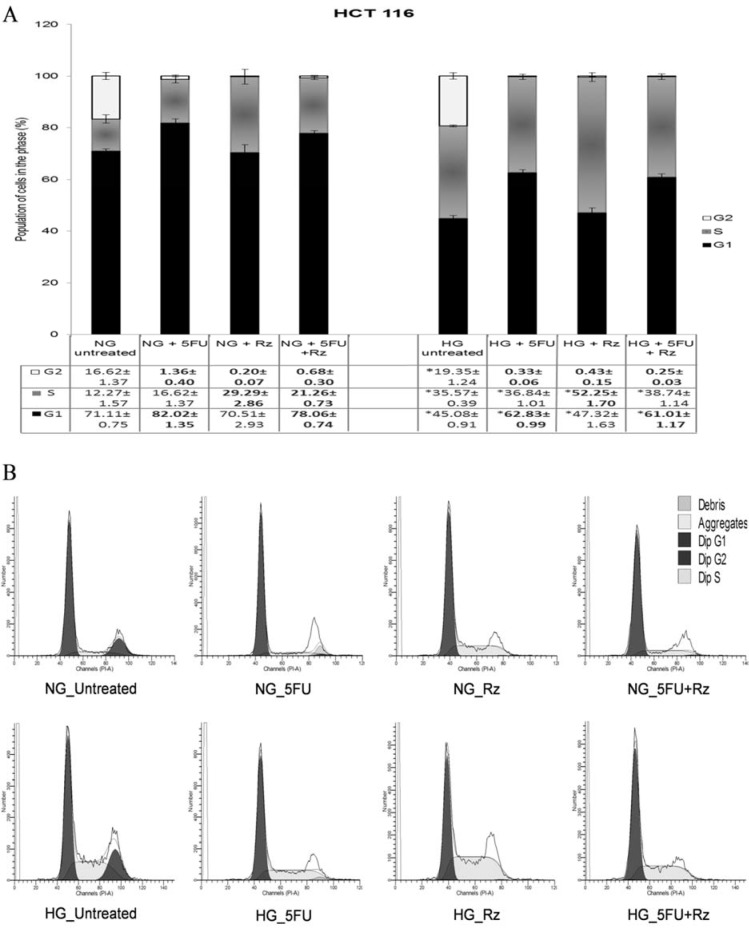
Figure 4: 5-FU pretreated cells (HCT 116) were exposed to different drug treatments for 48 h incubation in the present of normal glucose (NG) or high glucose (HG). (A) Data are presented as mean±SEM from three independent experiments in at least three replicates. Numbers in bold are significantly different compared to the values of untreated cells, p <0.05. (B) Representative DNA content histograms analyzed by Modfit software. *Compared to NG group with the same treatment, p <0.05. NG, normal glucose (5.5 mM); HG, high glucose (25 mM) Concentration of 5-FU= 0.2 µg/ml; Concentration of rosiglitazone= 0.5 µg/ml G1: Interphase Gap1; S: Interphase Synthesis; G2: Interphase Gap2

Exposure to low dose 5-FU caused G1 phase arrest on HCT 116 cells cultured in NG (82.02±1.35 %) and HG (62.83±0.99 %) conditions (Figure 4(Fig. 4)). However, low dose rosiglitazone arrested the cells in S phase in HG culture (52.25±1.70 %). In the presence of both 5-FU and rosiglitazone, G1 phase arrest was observed in NG (78.06±0.74 %) with S-phase cells significantly (p< 0.05) increased from 12.27±1.57 % to 21.26±0.73 % and HG (61.01±1.17 %) cultures. Under the similar treatments, HG culture significantly increased the cell proportion in S phase when compared to those in NG culture.

**Figure 5:**
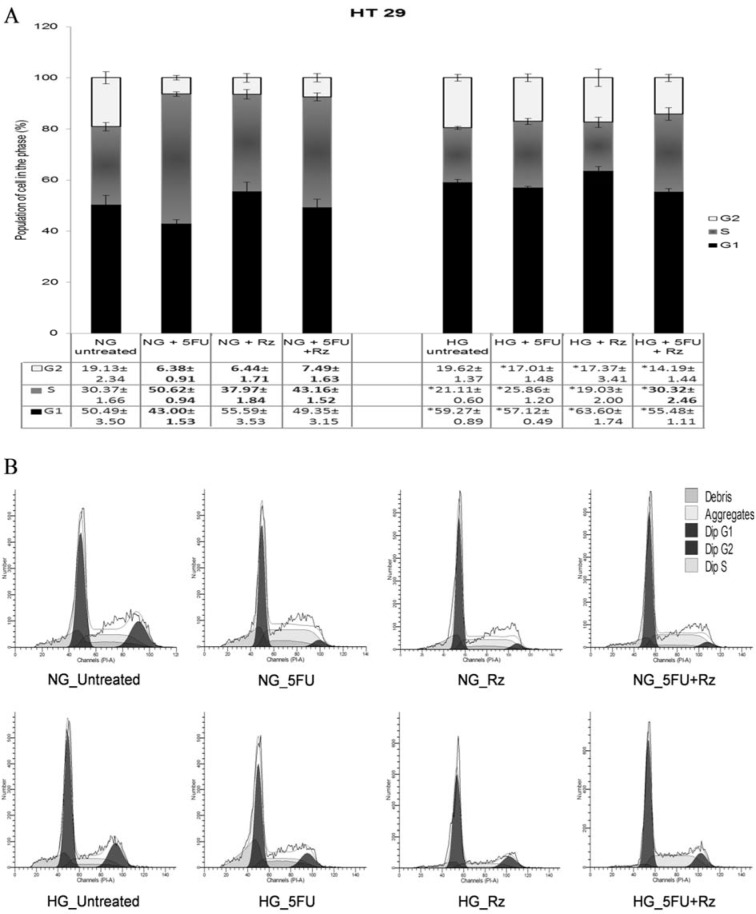
5-FU pretreated cells (HT 29) were exposed to different drug treatments for 48 h incubation in the present of normal glucose (NG) or high glucose (HG). (A) Data are presented as mean±SEM from three independent experiments in at least three replicates. Numbers in bold are significantly different compared to the values of untreated cells, p <0.05. (B) Representative DNA content histograms analyzed by Modfit software. *Compared to NG group with the same treatment, p <0.05. NG, normal glucose (5.5 mM); HG, high glucose (25 mM) Concentration of 5-FU= 0.2 µg/ml; Concentration of rosiglitazone= 0.5 µg/ml G1: Interphase Gap1; S: Interphase Synthesis; G2: Interphase Gap2

In NG culture, HT 29 cells treated with low dose 5-FU were arrested in S phase (50.62±0.94 %) as shown in Figure 5(Fig. 5). Low dose of rosiglitazone induced G1 phase arrest but significantly (p< 0.05) increased the proportion of S phase-cells from 30.37±1.66 % to 37.97±1.84 %. Treatment with 5-FU or rosiglitazone had no effect on the cell cycle in HG culture. The significant (p< 0.05) increase in cell proportion, 43.16±1.52 % (NG) and 30.32±2.46 % (HG), was observed in S phase when treated with both drugs together. Regardless of drug treatment, HG culture significantly (p< 0.05) increased the cell proportion in G2 phase when compared with NG culture.

## Discussion

Warburg hypothesis postulates that the origin of carcinogenesis is a change of metabolism which is often associated with enhanced glucose uptake and consumption in cancer cells (vander Heiden et al., 2009[43]). In this respect, hyperglycemia can be a primary glucose source to meet the metabolic demand of fast growing cancerous cells. Several *in vitro* studies have demonstrated that high glucose levels promote the proliferation of human colorectal carcinomas (Ma et al., 2014[30]; Masur et al., 2011[32]; Tomas et al., 2012[40]) which concur with the result obtained in this study. The increase in cell proliferation could be independent of osmotic stress as it was reproduced by D-mannitol with a significant difference against normal glucose. It was differed to normal cells which had no respond to glucose level (Figure 2A_3_(Fig. 2)).

5-FU is widely used to treat metastatic colorectal cancer. It is a uracil analog and principally works through irreversible inhibition of thymidylate synthase (TS), an enzyme responsible for the conversion of deoxyuridylate (dUMP) to deoxythymidylate (dTMP) under normal physiological conditions (Zhang et al., 2008[48]). 5-FU is converted to fluorodeoxyuridine monophosphate (FdUMP) which competes with dUMP in binding to TS and thus limiting dTMP production. The dTMP depletion can lead to cytotoxicity causing cell death via thymineless death (Longley et al., 2003[28]). Although cytotoxicity of 5-FU in colorectal cell lines have been extensively determined (Failli et al., 2011[10], 2013[11]; Flis and Spławiński, 2009[14]; Wiebke et al., 2003[45]), the role of glucose concentrations in altering the drug cytotoxicity still need to be clarified. In the present study, we showed that 5-FU and high glucose affected the cancer cell proliferation antagonistically.

As demonstrated in the study, the cytotoxicity of 5-FU in colorectal cancer cells was diminished by HG treatment, a concentration equivalent to the serum glucose level in diabetic individuals (glucose level >200 mg/dl). Conversely, proliferative effect of high glucose could be overcome when dosage of the drug was high enough. Ma et al. (2014[30]) speculated that a higher administration dosage is required to sustain the therapeutic effect of 5-FU for CRC patients with hyperglycemia. Unfortunately, cases of 5-FU toxicity have been reported on diabetic patients and the severity was directly related to the degree of hyperglycemia (Sadoff, 1998[37]). It implicated that administration of 5-FU can pose a threat to developing drug toxicity if blood glucose was poorly managed. Past clinical investigations have proposed that deficiency of, either dihydropyrimidine dehydrogenese or dihydropyrimidinase, is a pharmacogenetic disorder associated with 5-FU toxicity (Milano et al., 1999[34]; van Kuilenburg, 2004[41]; van Kuilenburg et al., 2003[42]), but the activities of these 5-FU catabolic enzymes in the cancer patients with diabetes are still not fully understood.

As the mainstay of chemotherapy for colorectal cancer, some common adverse events induced by 5-FU have been reported, namely mucositis, diarrhea and myelosuppression (Vincenzi et al., 2008[44]). 5-FU was also found to elevate blood glucose level in CRC patients (Köhne et al., 1997[22]; Tayek and Chlebowski, 1992[39]). A recent study even concluded that hyperglycemia is a potent complication due to 5FU-based regimen (Feng et al., 2013[12]). To address this issue, groups of antidiabetic agents including insulin analogs, insulin sensitizers, secretagogues and incretin mimitics are currently being considered. Based on a review by García-Jiménez et al. (2016[15]), agents that improve insulin sensitivity may reduce cancer risk rather than those that increase circulating insulin. Therefore, rosiglitazone as an insulin sensitizer should be appropriately applied in combination with 5-FU when taking cancer risk factors into consideration.

Ample evidences showed that rosiglitazone, a PPAR-gamma agonist suppressed proliferation of various cancerous cells at concentrations varying from 0.1 µmol/L to 100 µmol/L (Cao et al., 2009[4], 2015[5]; Han and Roman, 2006[18]; He et al., 2008[20]; Lin et al., 2007[25]; Zhang et al., 2008[49]). Similarly, in the present study, 5-FU pretreatment followed by a comparable dosage of rosiglitazone inhibited cell growth of HCT 116 and HT 29. While only one glucose concentration (16.67 mM or 11.11 mM) was used in the previous related studies (Lin et al., 2007[25]; Miao et al., 2011[33]; Zhang et al., 2007[51]), this study demonstrated that the inhibitory effect of rosiglitazone was modulated by glucose levels. Activation of PPAR-gamma by rosiglitazone stimulates the expression of phosphate and tension homolog (PTEN) in human carcinoma cell lines (Han and Roman, 2006[18]; Cao et al., 2009[4]). When glucose level is high, the PTEN expression is decreased leading to an increased Akt activity (Liu et al., 2012[27]; Mahimainathan et al., 2006[31]). The deregulation of Akt signaling allows cell survival and cell growth which may explain why the inhibitory effect of rosiglitazone was significantly (p< 0.05) reduced in HG culture (Figures 2C_1_-C_2_(Fig. 2)). There are other possible downstream regulations via a PPAR-gamma dependent signal pathway such as COX-2, MMP-7 and TIMP-1 (Miao et al., 2011[33]). 

5-FU treatment causes DNA damage due to misincorparation of FdUTP into DNA (Longley et al., 2003[28]). While cell cycle progression is regulated by checkpoint control in the G1 or G2 phase, cycle arrests in G1 and G2 phases allow DNA repair prior to replication and mitosis respectively. In the present study, low dose of 5-FU appeared to trigger cytostasis via G1 phase arrest on HCT 116 cells but S phase arrest on HT 29 cells in NG culture. The different cell cycle response might be due to the variance in mutation status of HCT 116 (MMR-deficient/p53-proficient) and HT 29 (MMR-proficient/p53-deficient). Admittedly, MMR-deficient cell lines confer less sensitivity to 5-FU (Adamsen et al., 2011[1]) thus, HG could diminish G1 phase arrest on 5FU-treated HCT 116 cells. The result was consistent with the increase of cell proliferation at high glucose level (Figure 2B_1_(Fig. 2)). On the other hand, Hawn et al. (1995[19]) suggested that any agent that induces DNA mispairs will lead to G2 arrest in MMR-proficient cells. The increase of HT 29 cells in G2 phase corresponded to the increase of cell proliferation (Figure 2B_2_(Fig. 2)) indicating that the mismatch repair (MMR) system interacted with G2 checkpoint in response to 5-FU, especially at high glucose level for DNA repair which allow cell replication. Unlike 5-FU, low dose rosiglitazone induced S-phase arrest and this differed to the effects whereby G1-phase arrest was evident on gastric cancer (He et al., 2008[20]), breast cancer (Zhang et al., 2008[49]), and liver cancer (Yu et al., 2010[47]). In fact, rosiglitazone was also reported to cause G1-phase arrest in colorectal cancers at higher concentrations specifically 10 µmol/L (HT 29) (Lin et al., 2007[25]) and 25 µmol/L (HCT 15) (Miao et al., 2011[33]). These conflicting results suggested that low dose of rosiglitazone might trigger the cells to experience genotoxic stress during DNA replication and delay their progression in a transient manner through activation of intra-S-phase checkpoint (Bartek et al., 2004[3]). Proteins that may be involved in the intra-S-phase checkpoint are kinase Chk1, MSH2 and MCH 1. It is possible to enhance the efficacy of 5-FU if DNA synthesis can be inhibited by regulating the TS levels during the S phase of cell cycle (Subbarayan et al., 2010[38]). The down-regulation of TS induces p53 protein expression (Liu et al., 2002[26]) and initiates cell apoptosis consequently.

To determine the drug interaction between rosiglitazone and 5-FU, the cytotoxicity outcomes from individual and combined drug treatments were further analyzed using combination index (CI) method (Chou and Talalay, 1983[6], 1984[7]). Based on CI values generated by CompuSyn software version 1.0 (data not shown), the present study revealed that high dose of rosiglitazone exerted synergistic effect on 5-FU treatment regardless of glucose levels. Indeed, it was far more effective than 5FU/metformin combination whereby the cell growth suppression occurred at a higher dosage of both drugs (Zhang et al., 2013[50]). Addition of low dose rosiglitazone was antagonistic to 5-FU, meaning that the combined drugs had an overall effect that was less than the sum of their individual effect. The cell cycle analysis reflected a similar trend between 5-FU treatment and combined drug treatment, whereby the highest cell proportion occurred in G1 phase followed by S phase and G2 phase under NG or HG culture.

## Conclusion

This study is the first attempt to demonstrate the influence of rosiglitazone on cytotoxicity of 5-FU under normal or high glucose condition. The present results showed that rosiglitazone had an antiproliferative effect on colorectal cancer cells via G1 or S phase arrest. Moreover, the antiproliferative effect was synergistic to 5-FU drug in the presence of high glucose. Thus, combining rosiglitazone in 5-FU regimen may improve the therapeutic effect. Taken together, the present finding provides a better insight for the management of hyperglycemic CRC patients on 5-FU chemotherapy. 

## Acknowledgement

This work was supported by University of Malaya Research Grant BK025-2014. 

## Declaration of interest

The authors disclose no conflicts of interest.
